# Preparing for an uncertain future: molecular responses of plants facing climate change

**DOI:** 10.1093/jxb/erac493

**Published:** 2022-12-14

**Authors:** Isabel Bäurle, Laurent Laplaze, Antoine Martin

**Affiliations:** Institute for Biochemistry and Biology, University of Potsdam, Potsdam, Germany; DIADE, Univ Montpellier, IRD, CIRAD, Montpellier, France; IPSiM, Univ Montpellier, CNRS, INRAE, Institut Agro, Montpellier, France; MPI of Molecular Plant Physiology, Germany

**Keywords:** Adaptation, climate change, drought, elevated CO_2_, heat stress, mitigation, resilience

**Climate change is mostly driven by the increase of atmospheric CO_2_ concentration. It leads to an increase in ambient temperature and temperature extremes, as well as to a reduction in water availability (Box 1). Despite the apparent benefit that an increase of CO_2_ and ambient temperature might have on the production of plant biomass, climate change severely challenges the plant life cycle, and thus increasingly leads to food insecurity. Investigating the molecular mechanisms of plant adaptation to climate change is thus a pressing issue. This was the theme of the EMBO Workshop ‘Molecular responses of plants facing climate change’ in June 2022 in Montpellier, France. Here, we summarize some key insights presented during the conference on the investigation of molecular mechanisms of plant adaptation to environmental constraints in a changing climate.**

**Box 1. The main factors of climate change influencing plant growth, development, and physiology**

CO_2_ concentration, temperature, and water availability are climate change-associated factors that are critical for plant growth and development. Climate change threatens both biodiversity and agriculture, with far-reaching implications for food security. Climate change is expected to reduce yield and to impact the quality of production, imposing major constraints on the higher demand of plant-based resources imposed by the growing population (Nelson, 2010). Thus, considerable research in the field focuses on understanding the physiological processes and underlying molecular mechanisms through which plants can adapt to these changes (Ahuja *et al.*, 2010; Nicotra *et al.*, 2010).

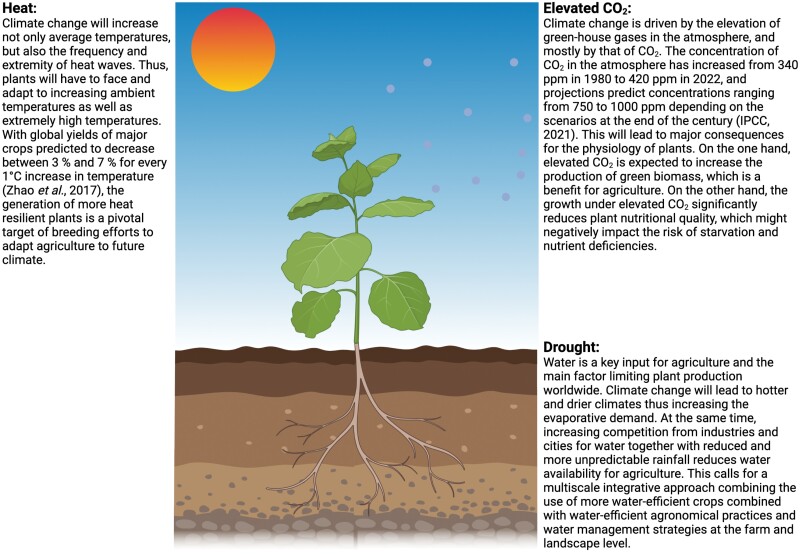

## How to deal with increasing temperatures?

Ambient temperatures affect all aspects of growth and development. Morphological responses to mildly elevated temperatures include the regulation of elongation growth in the shoot and the root (Quint *et al.*, 2016). In the shoot, several thermosensors including phytochrome B perceive temperature and coordinate with PHYTOCHROME-INTERACTING FACTOR 4 (PIF4) (Delker *et al.*, 2022; Perrella *et al.*, 2022). PIF4 then integrates with the auxin and brassinosteroid signalling pathways for a coherent growth response. Carolin Delker (University of Halle) discussed how shoot and root responses involve distinct sensing and signalling mechanisms. In nature, however, not only temperatures vary, but also light quality (shading) and water availability. Jorge Casal (University of Buenos Aires) highlighted the integration of different environmental cues at the level of photoreceptors and downstream signalling pathways to appropriately gauge complex environments (Casal and Balasubramanian, 2019; Romero-Montepaone *et al.*, 2020).

The heat stress response allows plants to acclimate to extreme temperatures through the activation of HEAT SHOCK TRANSCRIPTION FACTORs (HSFs), which are transcription factors that induce the expression of chaperones (Heat Shock Proteins, HSPs) and enzymes that produce thermoprotective and reactive oxygen species-scavenging metabolites (Ohama *et al.*, 2017; Guihur *et al.*, 2022). The current view is that HEAT SHOCK TRANSCRIPTION FACTOR A1 (HSFA1) isoforms are maintained in an inactive state in the cytoplasm through complexing with chaperones and rapidly move into the nucleus after heat stress to activate transcription, when the HSPs are recruited away from misfolded proteins (Ohama *et al.*, 2017). Phil Wigge (IGZ Großbeeren) presented data that suggest an alternative mode of regulation of at least one HSFA1 isoform by liquid–liquid phase separation. Intrinsically disordered domains within HSFA1 trigger phase separation at elevated temperatures, similar to what has been described for EARLY FLOWERING 3 (ELF3) (Jung *et al.*, 2020). Thus, HSFA1 may directly sense temperature. Temperature-dependent alternative splicing may constitute a parallel mechanism. Sotirios Fragkostefanakis (University of Frankfurt) reported on general splicing regulators with a specific role in heat stress-dependent alternative splicing, thus illustrating a feedback loop to fine-tune the heat stress response (Hu *et al.*, 2020; Rosenkranz *et al.*, 2022).

A moderate heat stress event can prime a plant and lead to a more efficient response upon recurrent stress after a stress-free interval lasting several days. This so-called heat stress memory shows complex regulation, including epigenetic modifications, protein stability and stable metabolites (Oberkofler *et al.*, 2021; Balazadeh, 2022). Isabel Bäurle (University of Potsdam) discussed epigenetic changes involving hyper-methylation of histone H3 lysine 4 (H3K4) and showed how locus-specific epigenetic editing of H3K4me3 impairs the memory (Oberkofler *et al.*, 2021; Oberkofler and Baurle, 2022). Specialized HSF complexes govern these epigenetic modifications (Friedrich *et al.*, 2021). In contrast, the autophagy cellular degradation machinery negatively impacts heat stress memory by degrading HSPs and other thermoprotective proteins, as Salma Balazadeh (University of Leiden) discussed (Thirumalaikumar *et al.*, 2021; Balazadeh, 2022). In a parallel pathway the metalloprotease FtsH6 degrades HSP21, thus modulating heat stress memory (Sedaghatmehr *et al.*, 2016). Improving heat stress memory provides an interesting avenue to increase tolerance to heat stress without affecting growth in stress-free conditions. Improvement of heat stress tolerance may also stem from clever manipulation of the soil microbiome. Heribert Hirt (King Abdullah University of Science and Technology) presented data showing that desert root endophytes induce thermotolerance by boosting heat stress memory (Shekhawat *et al.*, 2021).

## How to deal with water stress?

Root traits can be targeted to improve water capture in crops (Lynch, 2022) and tolerance to water stress based on a good characterization of the stress pattern and soil characteristics of the target area. Several mechanisms that optimize root development in response to changing soil water content (in time and space) are prime candidates. Xerobranching, the local inhibition of root branching when the root tip loses contact with wet soil (as in air gaps or dry soil regions), is an abscisic acid (ABA)-dependent adaptive process described in cereals and Arabidopsis (Orman-Ligeza *et al.*, 2018). Malcolm Bennett (University of Nottingham) described how a xerobranching water stress stimulus on a root tip leads to release of ABA from the phloem that induces closure of plasmodesmata in root outer tissues, which prevents the movement of auxin from the epidermis to the pericycle required for lateral root formation (Mehra *et al.*, 2022). This model explains how hydraulics controls root architecture via co-transport of phytohormones. Hydropatterning is another mechanism that regulates the radial positioning of lateral roots, root hairs, or aerenchyma depending on differences in water availability across the circumferential axis of the root to optimize root architecture to local water content (Bao *et al.*, 2014). This adaptive response is ABA-independent but dependent on sumoylation of AUXIN RESPONSE FACTOR 7 (ARF7), a key regulator of auxin response during lateral root formation (Orosa-Puente *et al.*, 2018). José Dinneny (Stanford University) reported that maize varieties are highly diverse for hydropatterning and that hydropatterning was a good predictor of root system depth, a trait associated with better water stress tolerance.

Integrating root architecture and anatomical and physiological properties of tissues is important to understand water dynamics in the root system (Maurel and Nacry, 2020). Christophe Maurel (Institute for Plant Sciences of Montpellier) showed by exploiting HydroRoot, a model for root hydraulics (Boursiac *et al.*, 2022), and split root experiments that different maize root types have different hydraulic properties and responses to water stress. He further reported genetic diversity in maize for root hydraulics. How root hydraulics relates to plant response to water stress still needs to be evaluated. Optimizing water acquisition by roots will increase water depletion in the soil and will ultimately have limited impact when available soil water is limited. Another complementary strategy is to improve water use efficiency, i.e. the amount of carbon fixed per unit water consumed. Transpiration is regulated by stomatal opening, a process that appeared in the earliest land plants (Clark *et al.*, 2022). Optimizing stomatal movement in response to environmental signals could improve plant water use efficiency and drought tolerance. Regulation of stomatal closure by ABA and CO_2_ is mediated by a complex signalling pathway involving several kinases (MPKs, OST1, HT1) and the GHR1 receptor-like pseudokinase, which was described by Hannes Kollist (University of Tartu) (Horak *et al.*, 2016; Sierla *et al.*, 2018). Stomata integrate environmental signals to optimize gas exchange and water loss. These signalling pathways ultimately lead to the coordinated regulation of different ion transporters localized at the plasma or vacuolar membrane of guard cells (Cubero-Font and De Angeli, 2021) as well as aquaporins of the PLASMA MEMBRANE INTRINSIC PROTEIN (PIP) subfamily (Ding *et al.*, 2022). The ability of the plant to restrict its transpiration when the evaporative demand is very high, and therefore when the water cost of carbon fixation is elevated, is a key trait for water use efficiency (Burridge *et al.*, 2022). Pablo Affortit (IRD) presented data showing a link between transpiration efficiency and transpiration restriction under high vapour pressure deficit on the one hand and plant hydraulics and root traits (in particular the root/shoot ratio) on the other in African rice (Affortit *et al.*, 2022), thus linking transpiration regulation to root water uptake. Interestingly, during drought stress, the photosynthetic apparatus is protected by a feedback control by photorespiration (Leverne and Krieger-Liszkay, 2021) and Anja Krieger-Liszkay (Institute for Integrative Biology of the Cell) presented evidence that signals deriving from the plastoquinone pool could regulate root growth. Altogether, this suggests mechanisms that integrate water stress perception in the photosynthetic tissues with root development and water uptake. Further work is needed to decipher the regulation of transpiration by plant hydraulics and photosynthesis.

## The increase of CO_2_ in the atmosphere: benefits, costs, and opportunities

Elevated CO_2_ concentration (eCO_2_) promotes the production of biomass, as the current CO_2_ concentration is a limiting factor for photosynthesis of C_3_ plants (Ainsworth and Long, 2021). As mentioned by Christine Foyer (University of Birmingham), this is a favourable circumstance in a world with a growing population. However, this CO_2_ fertilization effect is less than expected due to the acclimation of photosynthesis to eCO_2_. Boosting photosynthesis of C_3_ plants and understanding the mechanisms behind eCO_2_ acclimation in a CO_2_-enriched world are therefore main objectives for plant scientists (Simkin *et al.*, 2019). Different routes to improve photosynthesis were discussed during this workshop. One example was presented by Alejandro Perdomo (Lancaster University), who showed that the transcriptional regulation of Rubisco activase can be a way to improve Rubisco efficiency, and thus to increase carbon fixation and yield (Perdomo *et al.*, 2017). Florian Busch (University of Birmingham) discussed the different limitations of CO_2_ assimilation, and highlighted that the most relevant targets for improving photosynthesis vary depending on the environment. Together with Arnold Bloom (UC Davis), Busch stressed the importance of photorespiration, and challenged the view that this metabolic pathway would be a cost for plants compared with the improvement of photosynthesis (Busch *et al.*, 2018; Shi and Bloom, 2021).

However, the increase in carbon uptake and biomass production will not occur independently of other major metabolic pathways. This seems especially true considering that photosynthesis might not be the main limiting parameter for growth and yield, in opposition to nutrient and especially nitrogen limitation (Sinclair *et al.*, 2019). This is reminiscent of an unexpected negative effect of eCO_2_ on plants. Indeed, growth under eCO_2_ leads to a decline in the mineral composition of C_3_ plants, which might create a serious threat for food and nutritional security at the end of this century (Gojon *et al.*, 2022). Different approaches to identify the genetic and molecular mechanisms behind the negative effect of eCO_2_ on plant nutrient content have been presented. Antoine Martin (Institute for Plant Sciences of Montpellier) showed that the use of natural genetic variation or the inference of gene networks under simulated future climate notably led to the identification of genes involved in the response of plants to eCO_2_. Arnold Bloom presented data showing yield and protein content of several wheat lines over 35 years of cultivation in California. During this period CO_2_ increased in the atmosphere, while yield decreased but not protein content, suggesting that plants sacrificed yield to maintain organic N (Bloom and Plant, 2021). This also suggests that fast adaptive mechanisms to the changing atmosphere might occur, but remain to be identified.

Plants are also an extraordinary sink of carbon. The increase of CO_2_ in the atmosphere may lead to an augmentation in plant biomass, making plants a natural trap for atmospheric CO_2_ (Lynch, 2022) (Box 2). This was the subject of the keynote given by Joanne Chory (The Salk Institute), who presented data showing how root systems can be improved to capture and store carbon stably in the ground, contributing to the mitigation of climate change.

**Box 2. Some future challenges for plant science in the context of climate change**

Despite the progress already made, many challenges lie ahead: in many cases it is still an open question whether the mechanisms identified in one developmental stage are transferable to other developmental stages within one species. Moreover, whether the mechanisms outlined above and unraveled mostly in model plants are conserved in crop species remains an important question to investigate. Devising better adapted crops will require a good understanding of the stress pattern faced by crops in the target environments, a process that will benefit from the recent advances in crop modelling (Burridge *et al.*, 2022).

The emission of greenhouse gases (notably nitrous oxide coming indirectly from N fertilizers and methane from livestock), the use of fossil fuels (including for the production of N fertilizers), and intensive irrigation make agriculture a major contributor to climate change. Conversely, plant growth has been proposed as a solution to mitigate greenhouse gas emissions and the associated climate change effects. Indeed, by their ability to fix C and produce biomass, plants are an ideal tool to capture and to sequester atmospheric CO_2_ either as biomass or in soils. The worldwide distribution of crops makes them an excellent system to deploy a C-sequestration plan at a large scale. However, we still need to identify the best root traits to mobilize to achieve this goal and identify how this will impact the carbon dynamics in the soil in realistic (field) conditions. In addition, the development of root systems capable of taking up nutrients and water more efficiently from the soil is a way to reduce the use of N fertilizers and water, thereby considerably reducing the impact of agriculture on climate. Therefore, comprehension of the mechanisms driving root system development and N and water use efficiency under climate change is a major challenge to produce climate-resilient crops. Much remains to be understood in order to develop and use such climate-resilient plants in the near future (Dhankher and Foyer, 2018; Rivero *et al.*, 2022).

Ultimately, developing strategies to adapt agriculture to and mitigate the effects of climate change is a challenge that will require a truly interdisciplinary effort. While plant breeding will be a key component, sustainable solutions are unlikely to arise just from one discipline and will depend on system thinking. Plant biologists need to team up with other scientists such as soil scientists, agronomists, hydrologists, and economists to address these pressing issues.

## Overall conclusion

The complex nature of a changing climate requires plants to be systemically more efficient in dealing with the constraints that have been discussed here individually. A more integrated view and approach is required, as a plant that is more efficient in dealing with temperature extremes may at the same time be more vulnerable at elevated ambient temperature, under drought stress, or in a CO_2_-rich environment. This complexity calls for multi-partner initiatives that have the expertise and the means to tackle systemic aspects, ideally not in model plants, but directly in crop species.

## References

[CIT0001] Affortit P , Effa-Effa B, Ndoye MS, et al. 2022. Physiological and genetic control of transpiration efficiency in African rice, *Oryza glaberrima* Steud. Journal of Experimental Botany 73, 5279–5293. 35429274 10.1093/jxb/erac156

[CIT0002] Ahuja I , de Vos RC, Bones AM, Hall RD. 2010. Plant molecular stress responses face climate change. Trends in Plant Science 15, 664–674. 20846898 10.1016/j.tplants.2010.08.002

[CIT0003] Ainsworth EA , Long SP. 2021. 30 years of free-air carbon dioxide enrichment (FACE): what have we learned about future crop productivity and its potential for adaptation? Global Change Biology 27, 27–49. 33135850 10.1111/gcb.15375

[CIT0004] Balazadeh S. 2022. A ‘hot’ cocktail: the multiple layers of thermomemory in plants. Current Opinion in Plant Biology 65, 102147. 34861588 10.1016/j.pbi.2021.102147

[CIT0005] Bao Y , Aggarwal P, Robbins NE 2nd, et al. 2014. Plant roots use a patterning mechanism to position lateral root branches toward available water. Proceedings of the National Academy of Sciences, USA 111, 9319–9324. 10.1073/pnas.1400966111 PMC4078807 24927545

[CIT0006] Bloom AJ , Plant RE. 2021. Wheat grain yield decreased over the past 35 years, but protein content did not change. Journal of Experimental Botany 72, 6811–6821. 34318881 10.1093/jxb/erab343

[CIT0007] Boursiac Y , Pradal C, Bauget F, Lucas M, Delivorias S, Godin C, Maurel C. 2022. Phenotyping and modeling of root hydraulic architecture reveal critical determinants of axial water transport. Plant Physiology 190, 1289–1306. 35708646 10.1093/plphys/kiac281 PMC9516777

[CIT0008] Burridge JD , Grondin A, Vadez V. 2022. Optimizing crop water use for drought and climate change adaptation requires a multi-scale approach. Frontiers in Plant Science 13, 824720. 35574091 10.3389/fpls.2022.824720 PMC9100818

[CIT0009] Busch FA , Sage RF, Farquhar GD. 2018. Plants increase CO_2_ uptake by assimilating nitrogen via the photorespiratory pathway. Nature Plants 4, 46–54. 29229957 10.1038/s41477-017-0065-x

[CIT0010] Casal JJ , Balasubramanian S. 2019. Thermomorphogenesis. Annual Review in Plant Biology 70, 321–346. 10.1146/annurev-arplant-050718-095919 30786235

[CIT0011] Clark JW , Harris BJ, Hetherington AJ, Hurtado-Castano N, Brench RA, Casson S, Williams TA, Gray JE, Hetherington AM. 2022. The origin and evolution of stomata. Current Biology 32, R539–R553. 35671732 10.1016/j.cub.2022.04.040

[CIT0012] Cubero-Font P , De Angeli A. 2021. Connecting vacuolar and plasma membrane transport networks. New Phytologist 229, 755–762. 33007120 10.1111/nph.16983

[CIT0013] Delker C , Quint M, Wigge PA. 2022. Recent advances in understanding thermomorphogenesis signaling. Current Opinion in Plant Biology 68, 102231. 35636376 10.1016/j.pbi.2022.102231

[CIT0014] Dhankher OP , Foyer CH. 2018. Climate resilient crops for improving global food security and safety. Plant, Cell & Environment 41, 877–884. 10.1111/pce.13207 29663504

[CIT0015] Ding L , Milhiet T, Parent B, Meziane A, Tardieu F, Chaumont F. 2022. The plasma membrane aquaporin ZmPIP2;5 enhances the sensitivity of stomatal closure to water deficit. Plant, Cell & Environment 45, 1146–1156. 10.1111/pce.14276 35112729

[CIT0016] Friedrich T , Oberkofler V, Trindade I, et al. 2021. Heteromeric HSFA2/HSFA3 complexes drive transcriptional memory after heat stress in Arabidopsis. Nature Communications 12, 3426. 10.1038/s41467-021-23786-6 PMC8187452 34103516

[CIT0017] Gojon A , Cassan O, Bach L, Lejay L, Martin A. 2022. The decline of plant mineral nutrition under rising CO_2_: physiological and molecular aspects of a bad deal. Trends in Plant Science. doi: 10.1016/j.tplants.2022.09.002. 36336557

[CIT0018] Guihur A , Rebeaud ME, Goloubinoff P. 2022. How do plants feel the heat and survive? Trends in Biochemical Science 47, 824–838. 10.1016/j.tibs.2022.05.004 35660289

[CIT0019] Horak H , Sierla M, Toldsepp K, et al. 2016. A dominant mutation in the HT1 kinase uncovers roles of MAP kinases and GHR1 in CO_2_-induced stomatal closure. The Plant Cell 28, 2493–2509. 27694184 10.1105/tpc.16.00131 PMC5134974

[CIT0020] Hu Y , Mesihovic A, Jimenez-Gomez JM, Roth S, Gebhardt P, Bublak D, Bovy A, Scharf KD, Schleiff E, Fragkostefanakis S. 2020. Natural variation in HsfA2 pre-mRNA splicing is associated with changes in thermotolerance during tomato domestication. New Phytologist 225, 1297–1310. 31556121 10.1111/nph.16221

[CIT0021] IPCC. 2021. Climate change 2021: the physical science basis. Contribution of Working Group I to the Sixth Assessment Report of the Intergovernmental Panel on Climate Change. Masson-Delmotte V, Zhai P, Pirani A, et al., eds. Cambridge, New York: Cambridge University Press.

[CIT0022] Jung JH , Barbosa AD, Hutin S, et al. 2020. A prion-like domain in ELF3 functions as a thermosensor in *Arabidopsis*. Nature 585, 256–260. 32848244 10.1038/s41586-020-2644-7

[CIT0023] Leverne L , Krieger-Liszkay A. 2021. Moderate drought stress stabilizes the primary quinone acceptor QA and the secondary quinone acceptor QB in photosystem II. Physiologia Plantarum 171, 260–267. 33215720 10.1111/ppl.13286

[CIT0024] Lynch JP. 2022. Harnessing root architecture to address global challenges. The Plant Journal 109, 415–431. 34724260 10.1111/tpj.15560 PMC9299910

[CIT0025] Maurel C , Nacry P. 2020. Root architecture and hydraulics converge for acclimation to changing water availability. Nature Plants 6, 744–749. 32601421 10.1038/s41477-020-0684-5

[CIT0026] Mehra P , Pandey BK, Melebari D, et al. 2022. Hydraulic flux-responsive hormone redistribution determines root branching. Science 378, 762–768. 36395221 10.1126/science.add3771

[CIT0027] Nelson GC. 2010. The perfect storm. Significance 7, 13–16.

[CIT0028] Nicotra AB , Atkin OK, Bonser SP, et al. 2010. Plant phenotypic plasticity in a changing climate. Trends in Plant Science 15, 684–692. 20970368 10.1016/j.tplants.2010.09.008

[CIT0029] Oberkofler V , Baurle I. 2022. Inducible epigenome editing probes for the role of histone H3K4 methylation in Arabidopsis heat stress memory. Plant Physiology 189, 703–714. 35285498 10.1093/plphys/kiac113 PMC9157090

[CIT0030] Oberkofler V , Pratx L, Baurle I. 2021. Epigenetic regulation of abiotic stress memory: maintaining the good things while they last. Current Opinion in Plant Biology 61, 102007. 33571730 10.1016/j.pbi.2021.102007 PMC8250047

[CIT0031] Ohama N , Sato H, Shinozaki K, Yamaguchi-Shinozaki K. 2017. Transcriptional regulatory network of plant heat stress response. Trends in Plant Science 22, 53–65. 27666516 10.1016/j.tplants.2016.08.015

[CIT0032] Orman-Ligeza B , Morris EC, Parizot B, et al. 2018. The xerobranching response represses lateral root formation when roots are not in contact with water. Current Biology 28, 3165–3173.e5. 30270188 10.1016/j.cub.2018.07.074

[CIT0033] Orosa-Puente B , Leftley N, von Wangenheim D, et al. 2018. Root branching toward water involves posttranslational modification of transcription factor ARF7. Science 362, 1407–1410. 30573626 10.1126/science.aau3956

[CIT0034] Perdomo JA , Capó-Bauçà S, Carmo-Silva E, Galmés J. 2017. Rubisco and rubisco activase play an important role in the biochemical limitations of photosynthesis in rice, wheat, and maize under high temperature and water deficit. Frontiers in Plant Science 8, 490. 28450871 10.3389/fpls.2017.00490 PMC5390490

[CIT0035] Perrella G , Baurle I, van Zanten M. 2022. Epigenetic regulation of thermomorphogenesis and heat stress tolerance. New Phytologist 234, 1144–1160. 35037247 10.1111/nph.17970

[CIT0036] Quint M , Delker C, Franklin KA, Wigge PA, Halliday KJ, van Zanten M. 2016. Molecular and genetic control of plant thermomorphogenesis. Nature Plants 2, 15190. 27250752 10.1038/nplants.2015.190

[CIT0037] Rivero RM , Mittler R, Blumwald E, Zandalinas SI. 2022. Developing climate-resilient crops: improving plant tolerance to stress combination. The Plant Journal 109, 373–389. 34482588 10.1111/tpj.15483

[CIT0038] Romero-Montepaone S , Poodts S, Fischbach P, Sellaro R, Zurbriggen MD, Casal JJ. 2020. Shade avoidance responses become more aggressive in warm environments. Plant, Cell & Environment 43, 1625–1636. 10.1111/pce.13720 31925796

[CIT0039] Rosenkranz RRE , Ullrich S, Lochli K, Simm S, Fragkostefanakis S. 2022. Relevance and regulation of alternative splicing in plant heat stress response: current understanding and future directions. Frontiers in Plant Science 13, 911277. 35812973 10.3389/fpls.2022.911277 PMC9260394

[CIT0040] Sedaghatmehr M , Mueller-Roeber B, Balazadeh S. 2016. The plastid metalloprotease FtsH6 and small heat shock protein HSP21 jointly regulate thermomemory in *Arabidopsis*. Nature Communications 7, 12439. 10.1038/ncomms12439 PMC5007455 27561243

[CIT0041] Shekhawat K , Saad MM, Sheikh A, Mariappan K, Al-Mahmoudi H, Abdulhakim F, Eida AA, Jalal R, Masmoudi K, Hirt H. 2021. Root endophyte induced plant thermotolerance by constitutive chromatin modification at heat stress memory gene loci. EMBO Reports 22, e51049. 33426785 10.15252/embr.202051049 PMC7926228

[CIT0042] Shi X , Bloom A. 2021. Photorespiration: the futile cycle? Plants 10, 908. 34062784 10.3390/plants10050908 PMC8147352

[CIT0043] Sierla M , Horak H, Overmyer K, et al. 2018. The receptor-like pseudokinase GHR1 is required for stomatal closure. The Plant Cell 30, 2813–2837. 30361234 10.1105/tpc.18.00441 PMC6305979

[CIT0044] Simkin AJ , López-Calcagno PE, Raines CA. 2019. Feeding the world: improving photosynthetic efficiency for sustainable crop production. Journal of Experimental Botany 70, 1119–1140. 30772919 10.1093/jxb/ery445 PMC6395887

[CIT0045] Sinclair TR , Rufty TW, Lewis RS. 2019. Increasing photosynthesis: unlikely solution for world food problem. Trends in Plant Science 24, 1032–1039. 31488354 10.1016/j.tplants.2019.07.008

[CIT0046] Thirumalaikumar VP , Gorka M, Schulz K, Masclaux-Daubresse C, Sampathkumar A, Skirycz A, Vierstra RD, Balazadeh S. 2021. Selective autophagy regulates heat stress memory in Arabidopsis by NBR1-mediated targeting of HSP90.1 and ROF1. Autophagy 17, 2184–2199. 32967551 10.1080/15548627.2020.1820778 PMC8496721

[CIT0047] Zhao C , Liu B, Piao S, et al. 2017. Temperature increase reduces global yields of major crops in four independent estimates. Proceedings of the National Academy of Sciences, USA 114, 9326–9331. 10.1073/pnas.1701762114 PMC5584412 28811375

